# Sequence‐Specific Primer Polymerase Chain Reaction Genotyping of the Human *IL33* Polymorphism rs1929992 (T > C) with Sanger Sequencing Validation

**DOI:** 10.1002/cpz1.70455

**Published:** 2026-08-31

**Authors:** Aléia Harumi Uchibaba Yamanaka, Guilherme Castellani, Matheus Braga, Josiane Bazzo de Alencar, Letícia Cristina de Almeida Silva, Quirino Alves de Lima Neto

**Affiliations:** ^1^ Department of Basic Health Sciences (DBS), Laboratory of Immunogenetics (Imunogen‐UEM) State University of Maringá Maringá Paraná Brazil; ^2^ Laboratory of Medical Investigation in Dermatology and Immunodeficiencies (LIM‐56), Hospital das Clínicas, Faculdade de Medicina da Universidade de São Paulo (HCFMUSP) University of São Paulo São Paulo São Paulo Brazil

**Keywords:** genotyping techniques, interleukin‐33, molecular diagnostic techniques, polymerase chain reaction, polymorphism, single nucleotide

## Abstract

Single‐nucleotide polymorphisms (SNPs) may influence gene expression and contribute to interindividual variability in immune responses and disease susceptibility. Interleukin‐33 (IL33), a cytokine involved in inflammation, immune regulation, epithelial barrier homeostasis, and tissue remodeling, has been implicated in allergic, autoimmune, inflammatory, and neoplastic disorders. The *IL33* rs1929992 (T > C) polymorphism, located in intron 3, has been associated with altered IL33 expression, with the T allele linked to increased cytokine production compared with the C allele. This article describes a standardized sequence‐specific primer polymerase chain reaction (PCR‐SSP) protocol for genotyping the human *IL33* rs1929992 polymorphism, with validation by Sanger sequencing. The protocol includes optimization of annealing temperature, MgCl_2_ concentration, primer balance, deoxynucleotide triphosphates (dNTPs), and DNA input to ensure amplification specificity and reproducibility. Allele‐specific amplification is achieved using forward primers specific for the T and C alleles in independent reactions and analyzed by agarose gel electrophoresis, with the *human growth hormone* (*HGH*) gene used as an internal control. This protocol provides a simple, reproducible, and accessible method for rs1929992 genotyping, suitable for population‐based and genetic association studies involving immune‐mediated and inflammatory diseases. © 2026 The Author(s). *Current Protocols* published by Wiley Periodicals LLC.

**Basic Protocol 1**: PCR‐SSP genotyping of *IL33* rs1929992

**Support Protocol 1**: Extraction of genomic DNA by salting‐out

## Introduction

Interleukin‐33 (IL33) is a member of the IL‐1 cytokine family that functions as an alarmin released upon tissue injury or cellular stress, contributing to the regulation of innate and adaptive immune responses. Through signaling via the ST2 receptor, IL33 participates in epithelial barrier maintenance, inflammation, tissue remodeling, and type 2 immunity and has been implicated in the pathogenesis of allergic, autoimmune, inflammatory, and neoplastic diseases. Genetic variation within the *IL33* gene may influence cytokine expression and modify disease susceptibility. Among these variants, the rs1929992 (T > C) single‐nucleotide polymorphism (SNP), located in intron 3 of *IL33*, has been associated with altered *IL33* expression, with studies suggesting greater cytokine production in carriers of the T allele compared with the C allele.

Despite its analytical accuracy, Sanger sequencing may be impractical for routine genotyping in large sample collections because of labor requirements, turnaround time, and per‐sample cost. Alternative approaches, including TaqMan allelic discrimination assays and high‐resolution melting (HRM) analysis, may improve throughput but generally rely on fluorescence‐based chemistry, real‐time PCR systems, or specialized melting‐analysis platforms that are not universally available in conventional molecular laboratories. In contrast, sequence‐specific primer polymerase chain reaction (PCR‐SSP) enables SNP discrimination through conventional end‐point PCR followed by agarose gel electrophoresis. Previous applications of PCR‐SSP for immune‐related SNP genotyping further support the feasibility of this strategy in standard molecular biology laboratories (Castellani et al., 2026).

PCR‐SSP is based on allele‐selective amplification, in which primer extension occurs only when the 3′‐terminal nucleotide is complementary to the target sequence. For IL33 rs1929992, allele‐specific forward primers targeting the T and C variants are used in independent reactions together with a common reverse primer, enabling selective detection of each allele. After agarose gel electrophoresis, genotype assignment is determined by the amplification profile: amplification exclusively in the T‐specific reaction indicates a T/T genotype, amplification exclusively in the C‐specific reaction indicates a C/C genotype, and amplification in both reactions identifies heterozygous T/C samples. Basic Protocol 1 describes allele‐specific amplification and genotype determination of rs1929992, and Support Protocol 1 details genomic DNA extraction by salting‐out.

## PCR‐SSP GENOTYPING OF *IL33* rs1929992

This protocol describes a sequence‐specific primer polymerase chain reaction (PCR‐SSP) approach for genotyping the human *IL33* polymorphism rs1929992 (T > C). Each sample is analyzed using two independent amplification reactions containing allele‐specific forward primers designed to selectively detect either the T or C variant (F3.1 for T and F3.2 for C), together with a common reverse primer (R3). Allele discrimination is achieved by positioning the variant nucleotide at the 3′ terminus of the allele‐specific forward primer, allowing amplification only when complete complementarity with the target allele is present. A fragment of the human growth hormone (*HGH*) gene is coamplified as an internal control to verify amplification efficiency. Under optimized reaction conditions, the assay produces a distinct allele‐specific 155‐bp fragment together with the internal control band upon agarose gel electrophoresis, enabling reliable discrimination between homozygous and heterozygous genotypes.

### Materials

2% sodium hypochlorite
70% (w/v) ethanol
H_2_O, nuclease‐free
5 U/µl *Taq* DNA polymerase, recombinant (Invitrogen, cat. no. 10342020), supplied with 10× PCR buffer (200 mM Tris·Cl, pH 8.4/500 mM KCl) and 50 mM MgCl_2_ (store at −20°C)
dNTP mix (Thermo Fisher Scientific, cat. no. R0181, or equivalent)
*TLR9* primers (see Table 1)
*HGH* primers (see Table 1)
Genomic DNA (30‐50 ng/µl; see Support Protocol 1)
Reagents for gel electrophoresis:
  Agarose (Invitrogen, cat. no. 16500500, or equivalent)
  SYBR Safe DNA gel stain (Invitrogen, cat. no. S33102)
  10× Tris/borate/EDTA (TBE) buffer (Thermo Fisher Scientific, cat. no. B52, or equivalent)
  DNA ladder, 100 bp (GeneRuler 100 bp DNA ladder, Thermo Fisher Scientific, cat. no. SM0241)
  5× loading dye (Promega, cat. no. G190A, or equivalent)

Dedicated hood for PCR
2‐ to 20‐µl, 20‐ to 200‐µl, and 100‐ to 1000‐µl adjustable micropipets and appropriate sterile‐filtered pipet tips
0.2‐ml PCR tubes or strips
Thermal cycler (e.g., Veriti 96‐Well Fast Thermal Cycler, or equivalent)
Agarose gel electrophoresis apparatus
Gel documentation system

**Table 1:** Primers

Primers	Sequence (5′→ 3′)	Amplicon (pb)
Forward F3.1	CATTTTCCCCCCAAATTTCAAT	
Forward F3.2	CATTTTCCCCCCAAATTTCAAC	155
Reverse R3	AAGTCATCATCAACTTGGAACC	
*HGH*‐F	TGCCTTCCCAACCATTCCCTTA	460
*HGH*‐R	TCACGGATTTCTGTTGTCTTTC

1 Decontaminate the PCR workspace and pipets using 2% sodium hypochlorite followed by 70% ethanol. Avoid direct application of hypochlorite to glass surfaces.
2 Expose the PCR hood to ultraviolet light for 20 min before reaction setup.
3 Label 0.2‐ml PCR tubes for each sample, including known genotype controls and a no‐template control (NTC).
4 Prepare two master mixes on ice: one for the T allele reaction and one for the C allele reaction. The final reaction volume for each PCR is 10 µl (9 µl master mix + 1 µl genomic DNA).
  a. T allele reaction (F3.1/R3)—per reaction:
    PCR buffer: 1×
    MgCl_2_: 1.0 mM
    dNTP mix: 0.2 mM
*IL33* F3.1 primer: 0.25 µM
*IL33* R3 primer: 0.25 µM
*HGH* forward primer: 0.1 µM
*HGH* reverse primer: 0.1 µM
*Taq* DNA polymerase: 0.5 U.
  b. C allele reaction (F3.2/R3)—per reaction:
    PCR buffer: 1×
    MgCl_2_: 1.5 mM
    dNTP mix: 0.2 mM
*IL33* F3.2 primer: 0.25 µM
*IL33* R3 primer: 0.25 µM
*HGH* forward primer: 0.1 µM
*HGH* reverse primer: 0.1 µM
*Taq* DNA polymerase: 0.5 U.
5 For each reaction, dispense 9 µl master mix into each tube and add 1 µl genomic DNA (30‐50 ng/µl).
6 Run the following PCR program for both allele‐specific reactions:

**Table:** 

Initial step:	15 min	95°C
30 cycles:	30 s	95°C
	30 s	62°C
	30 s	72°C
Final step:	10 min	72°C
Hold at 4°C.
7 Add 2.5 µl of 5× loading buffer to each reaction and load 6 µl of PCR product onto a 2% (w/v) agarose gel. Perform electrophoresis at 150 V, 300 mA, and 150 W for 10 min. Successful allele‐specific amplification is indicated by the presence of a 155‐bp amplicon, together with the internal control band. Interpret genotypes as described in the Understanding Results section (see Fig. 1).

**Figure 1:**
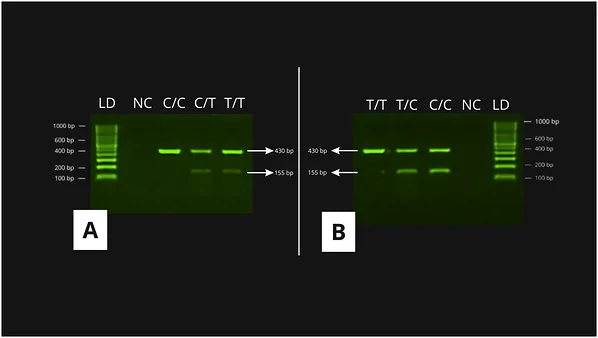
Representative agarose gel electrophoresis profile of PCR‐SSP products for *IL33* rs1929992 (T>C) genotyping. PCR amplicons were separated on a 2% agarose gel stained with SYBR Safe. (**A**) Amplification obtained using the T‐allele‐specific forward primer (F3.1). (**B**) Amplification obtained using the C‐allele‐specific forward primer (F3.2). LD indicates the 100‐bp DNA ladder. T/T, homozygous T; T/C, heterozygous; C/C, homozygous C; NTC, no‐template control. The 460‐bp fragment represents the coamplified *human growth hormone* (*HGH*) internal control, and the 155‐bp fragment corresponds to the rs1929992 allele‐specific amplification product. Sizes of DNA ladder fragments are shown in base pairs (bp) to verify expected amplicon size and electrophoretic migration.

## Support Protocol 1 GENOMIC DNA EXTRACTION FROM WHOLE BLOOD BY THE SALTING‐OUT METHOD

Genomic DNA is extracted from EDTA‐anticoagulated whole blood using the salting‐out method as originally described by John et al. (1991). This method provides a rapid, inexpensive, and organic‐solvent‐free approach for isolating high‐molecular‐weight genomic DNA suitable for downstream PCR‐based applications. The protocol described below follows the procedure reported in that study, with minor adaptations for sample volume and laboratory routine. All steps and reagent principles are derived from the original publication.

### Materials

Whole blood collected in EDTA tubes
Red blood cell lysis solution (10 mM Tris·Cl, pH 7.6/10 mM KCl/10 mM MgCl_2_; John et al., 1991)
Nucleus lysis buffer: 10 mM Tris·Cl, pH 8.2/400 mM NaCl/2 mM EDTA (see Current Protocols, 2006, for stock solutions)
Saturated NaCl solution
100% ethanol or isopropanol, ice cold
70% (v/v) ethanol
Nuclease‐free water or TE buffer (Current Protocols, 2006)

Microcentrifuge
1.5‐ml microcentrifuge tubes
2‐ to 20‐µl, 20‐ to 200‐µl, and 100‐ to 1000‐µl adjustable micropipets and appropriate sterile‐filtered pipet tips
Vortex mixer

Additional reagents and equipment for DNA quantification (Gallagher & Desjardins, 2008)

1 Lyse erythrocytes by adding 5 ml red blood cell lysis solution to 5 ml whole blood and mixing gently.
  This step removes erythrocytes while preserving leukocyte nuclei.
2 Centrifuge 10 min at ∼700 × *g*, room temperature, and discard the supernatant to obtain the leukocyte pellet.
3 Using a micropipet, resuspend the leukocyte pellet in 1 ml nucleus lysis buffer.
  This buffer stabilizes DNA and facilitates subsequent protein digestion.
4 Add 300 µl of saturated NaCl solution to the 1 ml of resuspended leukocytes and vortex vigorously to precipitate proteins.
  High salt concentration promotes aggregation and precipitation of proteins.
5 Centrifuge 3‐5 min at ≥10,000 × *g*, room temperature, and transfer the supernatant to a clean tube.
6 Precipitate DNA by adding cold 100% ethanol or isopropanol and gently inverting the tube.
7 Centrifuge to pellet DNA and discard supernatant.
8 Wash DNA pellet with 70% ethanol to remove residual salts.
9 Air‐dry the pellet briefly and resuspend in nuclease‐free water or TE buffer.
10 Quantify DNA concentration and store at −20°C until use in Basic Protocol.

## COMMENTARY

### Critical Parameters

#### Primer specificity

Allele discrimination in PCR‐SSP depends primarily on primer design. For *IL33* rs1929992, the polymorphic nucleotide must be positioned at the 3′ terminus of the allele‐specific forward primer (F3.1 for the T allele and F3.2 for the C allele). Because amplification occurs only when the terminal nucleotide is fully complementary to the target allele, even subtle mismatches or primer degradation may compromise specificity and result in weak or nonspecific amplification.

#### Annealing temperature

Using the optimized annealing temperature (62°C) is critical for maintaining selective amplification. Temperatures below this condition may increase nonspecific primer binding and false‐positive amplification, whereas higher temperatures may reduce amplification efficiency, particularly in samples with lower DNA concentration or quality.

#### MgCl_2_ concentration

Magnesium concentration strongly influences polymerase activity and primer‐template interactions. Optimal performance was achieved using 1.0 mM MgCl_2_ for the T allele reaction and 1.5 mM for the C allele reaction. Deviations from these concentrations may alter amplification specificity, band intensity, or reaction reproducibility.

#### DNA quality and concentration

High‐quality genomic DNA is required for reproducible amplification. DNA samples should ideally present an *A*_260_/*A*_280_ ratio of ∼1.8 and a concentration sufficient to provide 30‐50 ng DNA per reaction. Degraded DNA or residual contaminants may reduce amplification efficiency or produce inconsistent electrophoretic patterns.

### Troubleshooting

Common problems encountered during PCR‐SSP genotyping of *IL33* rs1929992 and their corresponding causes and solutions are summarized in Table 2.

**Table 2:** Troubleshooting Guide for PCR‐SSP Genotyping of *IL33* rs1929992

Problem	Possible cause	Solution
Amplification in NTC	Reagent contamination	Prepare fresh reagents; replace aliquots; physically separate pre‐ and post‐PCR areas.
No allele‐specific band (155 bp)	Low DNA quality or insufficient DNA	Verify DNA purity (*A*_260_/*A*_280_ ≈ 1.8) and concentration; repeat extraction if necessary.
Weak amplification	Suboptimal MgCl_2_ concentration	Reconfirm MgCl_2_ concentration according to allele‐specific reaction.
Nonspecific bands	Annealing temperature too low	Increase annealing temperature or verify primer specificity.

### Understanding Results

Representative electrophoretic patterns generated using the optimized PCR‐SSP assay for *IL33* rs1929992 (T>C) genotyping are presented in Figure 1. Successful reactions produce a 460‐bp fragment corresponding to the co‐amplified *human growth hormone* (*HGH*) internal control. Presence of this fragment confirms reaction integrity, adequate DNA quality, and proper PCR performance.

Allelic determination is based on amplification of a 155‐bp allele‐specific product. The T allele reaction, performed using the forward primer F3.1, produces amplification only when the rs1929992 T allele is present. Likewise, the C allele reaction, performed using primer F3.2, selectively amplifies samples carrying the C allele. Genotypes are assigned according to the following electrophoretic profile:

● 155‐bp band detected only in the T‐specific reaction → T/T genotype;
● 155‐bp band detected only in the C‐specific reaction → C/C genotype;
● 155‐bp band detected in both reactions → T/C genotype.

The *HGH* internal control band must be detected in every valid reaction. Samples lacking the internal control band should be considered inconclusive and reanalyzed before genotype assignment.

Figure 1A illustrates amplification profiles obtained in the T allele reaction, whereas Figure 1B shows amplification generated in the C allele reaction. Fragment size confirmation is provided by the 100‐bp DNA ladder (LD). No amplification should be observed in the no‐template control (NTC), confirming absence of reagent contamination.

After assay optimization, specific amplification was consistently achieved using 1.0 mM MgCl_2_ for the T allele reaction, 1.5 mM MgCl_2_ for the C allele reaction, 62°C annealing temperature, and 30‐50 ng genomic DNA per reaction. Under these conditions, expected fragments appear as distinct and well‐defined bands without detectable nonspecific amplification, facilitating straightforward genotype interpretation.

Analytical performance of the assay was assessed through comparison with Sanger sequencing, which demonstrated complete concordance with PCR‐SSP genotype assignments in representative validation samples. These findings support the reliability of allele‐specific amplification mediated by 3′‐terminal nucleotide complementarity.

When carried out under the conditions described here, this protocol provides consistent and reproducible genotyping results suitable for population‐based analyses and genetic association studies involving *IL33* rs1929992.

### Time Considerations

Genomic DNA extraction: ∼3‐4 h.

PCR amplification and electrophoresis: ∼4‐5 h.

Total time per batch of 24 samples: ∼1 working day.

### Author Contributions

**Aléia Harumi Uchibaba Yamanaka**: Conceptualization; investigation; writing—original draft; methodology; validation; writing—review and editing; visualization; data curation. **Guilherme Castellani**: Writing—original draft; writing—review and editing; investigation; methodology; data curation. **Matheus Braga**: Investigation; writing—review and editing; validation; formal analysis; data curation. **Josiane Bazzo de Alencar**: Investigation; writing—review and editing; writing—original draft; methodology; data curation. **Letícia Cristina de Almeida Silva**: Investigation; writing—review and editing; methodology; data curation. **Quirino Alves de Lima Neto**: Investigation; funding acquisition; writing—original draft; methodology; writing—review and editing; project administration; data curation; supervision; resources.

### Conflict of Interest

The authors declare no commercial or financial relationships that could be construed as a potential conflict of interest.

## Data Availability

The data, tools, and material (or their source) that support the protocol are available from the corresponding author upon reasonable request.
